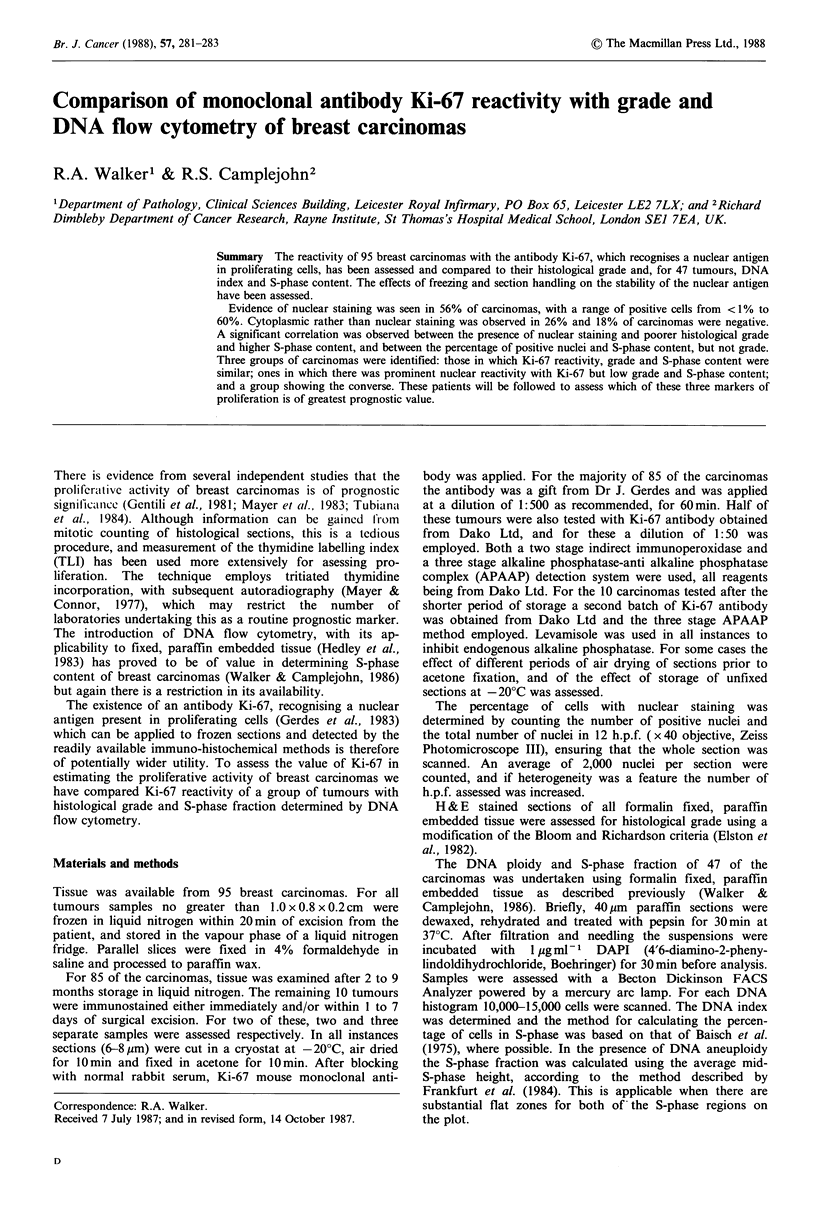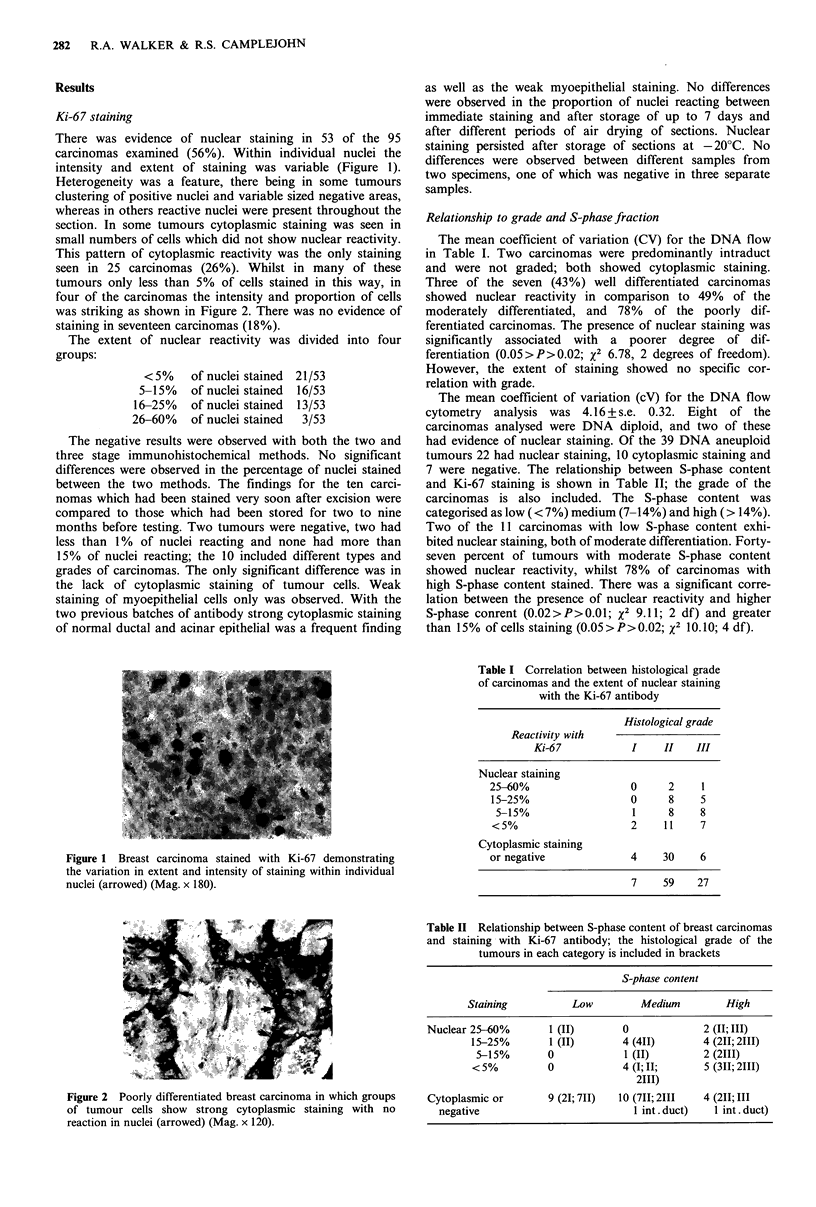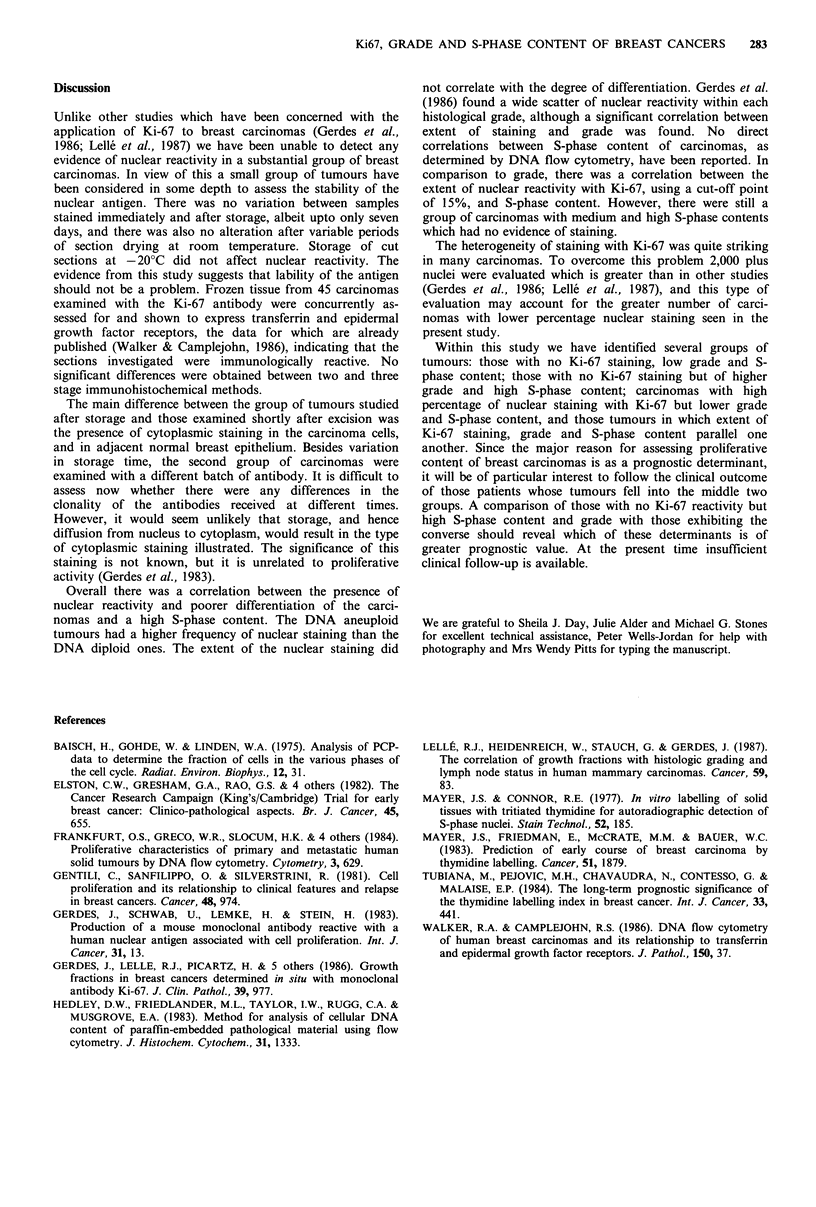# Comparison of monoclonal antibody Ki-67 reactivity with grade and DNA flow cytometry of breast carcinomas.

**DOI:** 10.1038/bjc.1988.60

**Published:** 1988-03

**Authors:** R. A. Walker, R. S. Camplejohn

**Affiliations:** Department of Pathology, Leicester Royal Infirmary, UK.

## Abstract

**Images:**


*Br. J. Cancer* (1988), **57**, 281–283 © The Macmillan Press Ltd., 1988

### Comparison of monoclonal antibody Ki-67 reactivity with grade and DNA flow cytometry of breast carcinomas

R.A. Walker[1] & R.S. Camplejohn[2]

[1]*Department of Pathology, Clinical Sciences Building, Leicester Royal Infirmary, PO Box 65, Leicester LE2 7LX; and* [2]*Richard Dimbleby Department of Cancer Research, Rayne Institute, St Thomas's Hospital Medical School, London SE1 7EA, UK.*

**Summary** The reactivity of 95 breast carcinomas with the antibody Ki-67, which recognises a nuclear antigen in proliferating cells, has been assessed and compared to their histological grade and, for 47 tumours, DNA index and S-phase content. The effects of freezing and section handling on the stability of the nuclear antigen have been assessed.

Evidence of nuclear staining was seen in 56% of carcinomas, with a range of positive cells from <1% to 60%. Cytoplasmic rather than nuclear staining was observed in 26% and 18% of carcinomas were negative. A significant correlation was observed between the presence of nuclear staining and poorer histological grade and higher S-phase content, and between the percentage of positive nuclei and S-phase content, but not grade. Three groups of carcinomas were identified: those in which Ki-67 reactivity, grade and S-phase content were similar; ones in which there was prominent nuclear reactivity with Ki-67 but low grade and S-phase content; and a group showing the converse. These patients will be followed to assess which of these three markers of proliferation is of greatest prognostic value.

There is evidence from several independent studies that the proliferative activity of breast carcinomas is of prognostic significance (Gentili *et al.*, 1981; Mayer *et al.*, 1983; Tubiana *et al.*, 1984). Although information can be gained from mitotic counting of histological sections, this is a tedious procedure, and measurement of the thymidine labelling index (TLI) has been used more extensively for asessing proliferation. The technique employs tritiated thymidine incorporation, with subsequent autoradiography (Mayer & Connor, 1977), which may restrict the number of laboratories undertaking this as a routine prognostic marker. The introduction of DNA flow cytometry, with its applicability to fixed, paraffin embedded tissue (Hedley *et al.*, 1983) has proved to be of value in determining S-phase content of breast carcinomas (Walker & Camplejohn, 1986) but again there is a restriction in its availability.

The existence of an antibody Ki-67, recognising a nuclear antigen present in proliferating cells (Gerdes *et al.*, 1983) which can be applied to frozen sections and detected by the readily available immuno-histochemical methods is therefore of potentially wider utility. To assess the value of Ki-67 in estimating the proliferative activity of breast carcinomas we have compared Ki-67 reactivity of a group of tumours with histological grade and S-phase fraction determined by DNA flow cytometry.

### Materials and methods

Tissue was available from 95 breast carcinomas. For all tumours samples no greater than $1.0 \times 0.8 \times 0.2$ cm were frozen in liquid nitrogen within 20 min of excision from the patient, and stored in the vapour phase of a liquid nitrogen fridge. Parallel slices were fixed in 4% formaldehyde in saline and processed to paraffin wax.

For 85 of the carcinomas, tissue was examined after 2 to 9 months storage in liquid nitrogen. The remaining 10 tumours were immunostained either immediately and/or within 1 to 7 days of surgical excision. For two of these, two and three separate samples were assessed respectively. In all instances sections (6–8 $\mu$m) were cut in a cryostat at $-20$°C, air dried for 10 min and fixed in acetone for 10 min. After blocking with normal rabbit serum, Ki-67 mouse monoclonal antibody was applied. For the majority of 85 of the carcinomas the antibody was a gift from Dr J. Gerdes and was applied at a dilution of 1:500 as recommended, for 60 min. Half of these tumours were also tested with Ki-67 antibody obtained from Dako Ltd, and for these a dilution of 1:50 was employed. Both a two stage indirect immunoperoxidase and a three stage alkaline phosphatase-anti alkaline phosphatase complex (APAAP) detection system were used, all reagents being from Dako Ltd. For the 10 carcinomas tested after the shorter period of storage a second batch of Ki-67 antibody was obtained from Dako Ltd and the three stage APAAP method employed. Levamisole was used in all instances to inhibit endogenous alkaline phosphatase. For some cases the effect of different periods of air drying of sections prior to acetone fixation, and of the effect of storage of unfixed sections at $-20$°C was assessed.

The percentage of cells with nuclear staining was determined by counting the number of positive nuclei and the total number of nuclei in 12 h.p.f. ($\times 40$ objective, Zeiss Photomicroscope III), ensuring that the whole section was scanned. An average of 2,000 nuclei per section were counted, and if heterogeneity was a feature the number of h.p.f. assessed was increased.

H&E stained sections of all formalin fixed, paraffin embedded tissue were assessed for histological grade using a modification of the Bloom and Richardson criteria (Elston *et al.*, 1982).

The DNA ploidy and S-phase fraction of 47 of the carcinomas was undertaken using formalin fixed, paraffin embedded tissue as described previously (Walker & Camplejohn, 1986). Briefly, 40 $\mu$m paraffin sections were dewaxed, rehydrated and treated with pepsin for 30 min at 37°C. After filtration and needling the suspensions were incubated with 1 $\mu$g ml$^{-1}$ DAPI (4′6-diamino-2-phenylindoldihydrochloride, Boehringer) for 30 min before analysis. Samples were assessed with a Becton Dickinson FACS Analyzer powered by a mercury arc lamp. For each DNA histogram 10,000–15,000 cells were scanned. The DNA index was determined and the method for calculating the percentage of cells in S-phase was based on that of Baisch *et al.* (1975), where possible. In the presence of DNA aneuploidy the S-phase fraction was calculated using the average mid-S-phase height, according to the method described by Frankfurt *et al.* (1984). This is applicable when there are substantial flat zones for both of the S-phase regions on the plot.

Correspondence: R.A. Walker.
Received 7 July 1987; and in revised form, 14 October 1987.

### Results

### *Ki-67 staining*

There was evidence of nuclear staining in 53 of the 95 carcinomas examined (56%). Within individual nuclei the intensity and extent of staining was variable (Figure 1). Heterogeneity was a feature, there being in some tumours clustering of positive nuclei and variable sized negative areas, whereas in others reactive nuclei were present throughout the section. In some tumours cytoplasmic staining was seen in small numbers of cells which did not show nuclear reactivity. This pattern of cytoplasmic reactivity was the only staining seen in 25 carcinomas (26%). Whilst in many of these tumours only less than 5% of cells stained in this way, in four of the carcinomas the intensity and proportion of cells was striking as shown in Figure 2. There was no evidence of staining in seventeen carcinomas (18%).

The extent of nuclear reactivity was divided into four groups:

| | | |
|---|---|---|
| <5% | of nuclei stained | 21/53 |
| 5–15% | of nuclei stained | 16/53 |
| 16–25% | of nuclei stained | 13/53 |
| 26–60% | of nuclei stained | 3/53 |

The negative results were observed with both the two and three stage immunohistochemical methods. No significant differences were observed in the percentage of nuclei stained between the two methods. The findings for the ten carcinomas which had been stained very soon after excision were compared to those which had been stored for two to nine months before testing. Two tumours were negative, two had less than 1% of nuclei reacting and none had more than 15% of nuclei reacting; the 10 included different types and grades of carcinomas. The only significant difference was in the lack of cytoplasmic staining of tumour cells. Weak staining of myoepithelial cells only was observed. With the two previous batches of antibody strong cytoplasmic staining of normal ductal and acinar epithelial was a frequent finding as well as the weak myoepithelial staining. No differences were observed in the proportion of nuclei reacting between immediate staining and after storage of up to 7 days and after different periods of air drying of sections. Nuclear staining persisted after storage of sections at $-20$°C. No differences were observed between different samples from two specimens, one of which was negative in three separate samples.

### *Relationship to grade and S-phase fraction*

The mean coefficient of variation (CV) for the DNA flow in Table I. Two carcinomas were predominantly intraduct and were not graded; both showed cytoplasmic staining. Three of the seven (43%) well differentiated carcinomas showed nuclear reactivity in comparison to 49% of the moderately differentiated, and 78% of the poorly differentiated carcinomas. The presence of nuclear staining was significantly associated with a poorer degree of differentiation ($0.05 > P > 0.02$; $\chi^2$ 6.78, 2 degrees of freedom). However, the extent of staining showed no specific correlation with grade.

The mean coefficient of variation (cV) for the DNA flow cytometry analysis was 4.16±s.e. 0.32. Eight of the carcinomas analysed were DNA diploid, and two of these had evidence of nuclear staining. Of the 39 DNA aneuploid tumours 22 had nuclear staining, 10 cytoplasmic staining and 7 were negative. The relationship between S-phase content and Ki-67 staining is shown in Table II; the grade of the carcinomas is also included. The S-phase content was categorised as low (<7%) medium (7–14%) and high (>14%). Two of the 11 carcinomas with low S-phase content exhibited nuclear staining, both of moderate differentiation. Forty-seven percent of tumours with moderate S-phase content showed nuclear reactivity, whilst 78% of carcinomas with high S-phase content stained. There was a significant correlation between the presence of nuclear reactivity and higher S-phase conrent ($0.02 > P > 0.01$; $\chi^2$ 9.11; 2 df) and greater than 15% of cells staining ($0.05 > P > 0.02$; $\chi^2$ 10.10; 4 df).

**Figure 1** Breast carcinoma stained with Ki-67 demonstrating the variation in extent and intensity of staining within individual nuclei (arrowed) (Mag. x 180).

**Figure 2** Poorly differentiated breast carcinoma in which groups of tumour cells show strong cytoplasmic staining with no reaction in nuclei (arrowed) (Mag. x 120).

**Table I** Correlation between histological grade of carcinomas and the extent of nuclear staining with the Ki-67 antibody

| *Reactivity with Ki-67* | *Histological grade* | | |
|---|---|---|---|
| | *I* | *II* | *III* |
| Nuclear staining | | | |
| 25–60% | 0 | 2 | 1 |
| 15–25% | 0 | 8 | 5 |
| 5–15% | 1 | 8 | 8 |
| <5% | 2 | 11 | 7 |
| Cytoplasmic staining or negative | 4 | 30 | 6 |
| | 7 | 59 | 27 |

**Table II** Relationship between S-phase content of breast carcinomas and staining with Ki-67 antibody; the histological grade of the tumours in each category is included in brackets

| *Staining* | *S-phase content* | | |
|---|---|---|---|
| | *Low* | *Medium* | *High* |
| Nuclear 25–60% | 1 (II) | 0 | 2 (II; III) |
| 15–25% | 1 (II) | 4 (4II) | 4 (2II; 2III) |
| 5–15% | 0 | 1 (II) | 2 (2III) |
| <5% | 0 | 4 (I; II; 2III) | 5 (3II; 2III) |
| Cytoplasmic or negative | 9 (2I; 7II) | 10 (7II; 2III 1 int. duct) | 4 (2II; III 1 int. duct) |

### Discussion

Unlike other studies which have been concerned with the application of Ki-67 to breast carcinomas (Gerdes *et al.*, 1986; Lellé *et al.*, 1987) we have been unable to detect any evidence of nuclear reactivity in a substantial group of breast carcinomas. In view of this a small group of tumours have been considered in some depth to assess the stability of the nuclear antigen. There was no variation between samples stained immediately and after storage, albeit upto only seven days, and there was also no alteration after variable periods of section drying at room temperature. Storage of cut sections at −20°C did not affect nuclear reactivity. The evidence from this study suggests that lability of the antigen should not be a problem. Frozen tissue from 45 carcinomas examined with the Ki-67 antibody were concurrently assessed for and shown to express transferrin and epidermal growth factor receptors, the data for which are already published (Walker & Camplejohn, 1986), indicating that the sections investigated were immunologically reactive. No significant differences were obtained between two and three stage immunohistochemical methods.

The main difference between the group of tumours studied after storage and those examined shortly after excision was the presence of cytoplasmic staining in the carcinoma cells, and in adjacent normal breast epithelium. Besides variation in storage time, the second group of carcinomas were examined with a different batch of antibody. It is difficult to assess now whether there were any differences in the clonality of the antibodies received at different times. However, it would seem unlikely that storage, and hence diffusion from nucleus to cytoplasm, would result in the type of cytoplasmic staining illustrated. The significance of this staining is not known, but it is unrelated to proliferative activity (Gerdes *et al.*, 1983).

Overall there was a correlation between the presence of nuclear reactivity and poorer differentiation of the carcinomas and a high S-phase content. The DNA aneuploid tumours had a higher frequency of nuclear staining than the DNA diploid ones. The extent of the nuclear staining did not correlate with the degree of differentiation. Gerdes *et al.* (1986) found a wide scatter of nuclear reactivity within each histological grade, although a significant correlation between extent of staining and grade was found. No direct correlations between S-phase content of carcinomas, as determined by DNA flow cytometry, have been reported. In comparison to grade, there was a correlation between the extent of nuclear reactivity with Ki-67, using a cut-off point of 15%, and S-phase content. However, there were still a group of carcinomas with medium and high S-phase contents which had no evidence of staining.

The heterogeneity of staining with Ki-67 was quite striking in many carcinomas. To overcome this problem 2,000 plus nuclei were evaluated which is greater than in other studies (Gerdes *et al.*, 1986; Lellé *et al.*, 1987), and this type of evaluation may account for the greater number of carcinomas with lower percentage nuclear staining seen in the present study.

Within this study we have identified several groups of tumours: those with no Ki-67 staining, low grade and S-phase content; those with no Ki-67 staining but of higher grade and high S-phase content; carcinomas with high percentage of nuclear staining with Ki-67 but lower grade and S-phase content, and those tumours in which extent of Ki-67 staining, grade and S-phase content parallel one another. Since the major reason for assessing proliferative content of breast carcinomas is as a prognostic determinant, it will be of particular interest to follow the clinical outcome of those patients whose tumours fell into the middle two groups. A comparison of those with no Ki-67 reactivity but high S-phase content and grade with those exhibiting the converse should reveal which of these determinants is of greater prognostic value. At the present time insufficient clinical follow-up is available.

We are grateful to Sheila J. Day, Julie Alder and Michael G. Stones for excellent technical assistance, Peter Wells-Jordan for help with photography and Mrs Wendy Pitts for typing the manuscript.

### References

BAISCH, H., GOHDE, W. & LINDEN, W.A. (1975). Analysis of PCP-data to determine the fraction of cells in the various phases of the cell cycle. *Radiat. Environ. Biophys.*, **12**, 31.

ELSTON, C.W., GRESHAM, G.A., RAO, G.S. & 4 others (1982). The Cancer Research Campaign (King's/Cambridge) Trial for early breast cancer: Clinico-pathological aspects. *Br. J. Cancer*, **45**, 655.

FRANKFURT, O.S., GRECO, W.R., SLOCUM, H.K. & 4 others (1984). Proliferative characteristics of primary and metastatic human solid tumours by DNA flow cytometry. *Cytometry*, **3**, 629.

GENTILI, C., SANFILIPPO, O. & SILVERSTRINI, R. (1981). Cell proliferation and its relationship to clinical features and relapse in breast cancers. *Cancer*, **48**, 974.

GERDES, J., SCHWAB, U., LEMKE, H. & STEIN, H. (1983). Production of a mouse monoclonal antibody reactive with a human nuclear antigen associated with cell proliferation. *Int. J. Cancer*, **31**, 13.

GERDES, J., LELLE, R.J., PICARTZ, H. & 5 others (1986). Growth fractions in breast cancers determined *in situ* with monoclonal antibody Ki-67. *J. Clin. Pathol.*, **39**, 977.

HEDLEY, D.W., FRIEDLANDER, M.L., TAYLOR, I.W., RUGG, C.A. & MUSGROVE, E.A. (1983). Method for analysis of cellular DNA content of paraffin-embedded pathological material using flow cytometry. *J. Histochem. Cytochem.*, **31**, 1333.

LELLÉ, R.J., HEIDENREICH, W., STAUCH, G. & GERDES, J. (1987). The correlation of growth fractions with histologic grading and lymph node status in human mammary carcinomas. *Cancer*, **59**, 83.

MAYER, J.S. & CONNOR, R.E. (1977). *In vitro* labelling of solid tissues with tritiated thymidine for autoradiographic detection of S-phase nuclei. *Stain Technol.*, **52**, 185.

MAYER, J.S., FRIEDMAN, E., McCRATE, M.M. & BAUER, W.C. (1983). Prediction of early course of breast carcinoma by thymidine labelling. *Cancer*, **51**, 1879.

TUBIANA, M., PEJOVIC, M.H., CHAVAUDRA, N., CONTESSO, G. & MALAISE, E.P. (1984). The long-term prognostic significance of the thymidine labelling index in breast cancer. *Int. J. Cancer*, **33**, 441.

WALKER, R.A. & CAMPLEJOHN, R.S. (1986). DNA flow cytometry of human breast carcinomas and its relationship to transferrin and epidermal growth factor receptors. *J. Pathol.*, **150**, 37.